# Multicenter evaluation of dynamic three-dimensional whole-heart myocardial perfusion imaging for the detection of coronary artery disease defined by fractional flow reserve

**DOI:** 10.1186/1532-429X-15-S1-O103

**Published:** 2013-01-30

**Authors:** Robert Manka, Rolf Gebker, Lukas Wissmann, Roy Jogiya, Manish Motwani, Michael Frick, Sebastian D Reinartz, Bernhard Schnackenburg, Eike Nagel, Sven Plein, Sebastian Kozerke

**Affiliations:** 1 University Hospital Zürich, Zürich, Switzerland; 2 Institute for Biomedical Engineering University and ETH zürich, Zürich, Switzerland; 3 German Heart Institute, Berlin, Germany; 4 King's College London, London, UK; 5 University of Leeds, Leeds, UK; 6 University Hospital RWTH Aachen, Aachen, Germany

## Background

Cardiac magnetic resonance (CMR) perfusion imaging yields high diagnostic accuracy for the detection of coronary artery disease (CAD) [1]. However, standard 2D multi-slice CMR perfusion techniques only provide limited coverage and hence prohibit computation of myocardial ischemic burden. Recently, two single-center 3D CMR perfusion studies have proven highly diagnostic for the detection of CAD relative to quantitative coronary angiography (QCA) [2] and fractional flow reserve (FFR) [3]. The aim of our prospective multicenter study is to assess the diagnostic performance of 3D CMR perfusion imaging in comparison with FFR.

## Methods

Five centers across Europe have been enrolled to acquire 3D CMR perfusion and FFR data in a total of 150 patients with suspected CAD. CMR 3D perfusion imaging employs 10-fold accelerated k-t PCA [4] providing whole heart coverage with a spatial resolution of 2.3x2.3x5 mm3 on 3.0T systems (Philips Healthcare, Best, The Netherlands). Perfusion scans were obtained under adenosine stress (140 μg/kg/min for 6 min; 0.075 mmol/kg gadobutrol; Gadovist, Bayer Schering Pharma, Berlin, Germany) and at rest. FFR was recorded in all patent epicardial coronary arteries (significant stenosis <0.75). For visual analysis, 3D CMR perfusion scans were classified as pathologic if ≥1 segment showed an inducible perfusion deficit with >25% transmurality. Overall image quality of stress and rest 3D CMR perfusion scans was graded on a scale between 1 and 4 (1= non-diagnostic, 2= poor, 3= good, 4= excellent). All CMR perfusion analyses were performed in central core-lab, blinded to all clinical data.

## Results

Here we report preliminary results obtained in the first 40 patients (mean age 63+/-11 years, 11 female). CAD prevalence as defined by FFR (<0.75) was 38% (15 of 40 patients). 3D CMR perfusion resulted in a sensitivity and specificity of 87% and 84%, respectively. The mean visual score of 3D perfusion imaging was 3.4 ± 0.7 during adenosine stress and 3.6 ± 0.5 at rest (p= 0.06). No study was graded as non-diagnostic.

## Conclusions

In this preliminary assessment of a sub cohort of our multicenter study 3D CMR perfusion imaging proved highly diagnostic for the detection of functionally significant CAD as defined by FFR. Analysis of the remaining data is required to fully conclude on the finding presented here.

## Funding

Swiss National Science Foundation, grant #CR3213_132671/1, Bayer Schering Pharma, Zürich Switzerland.

**Figure 1:**
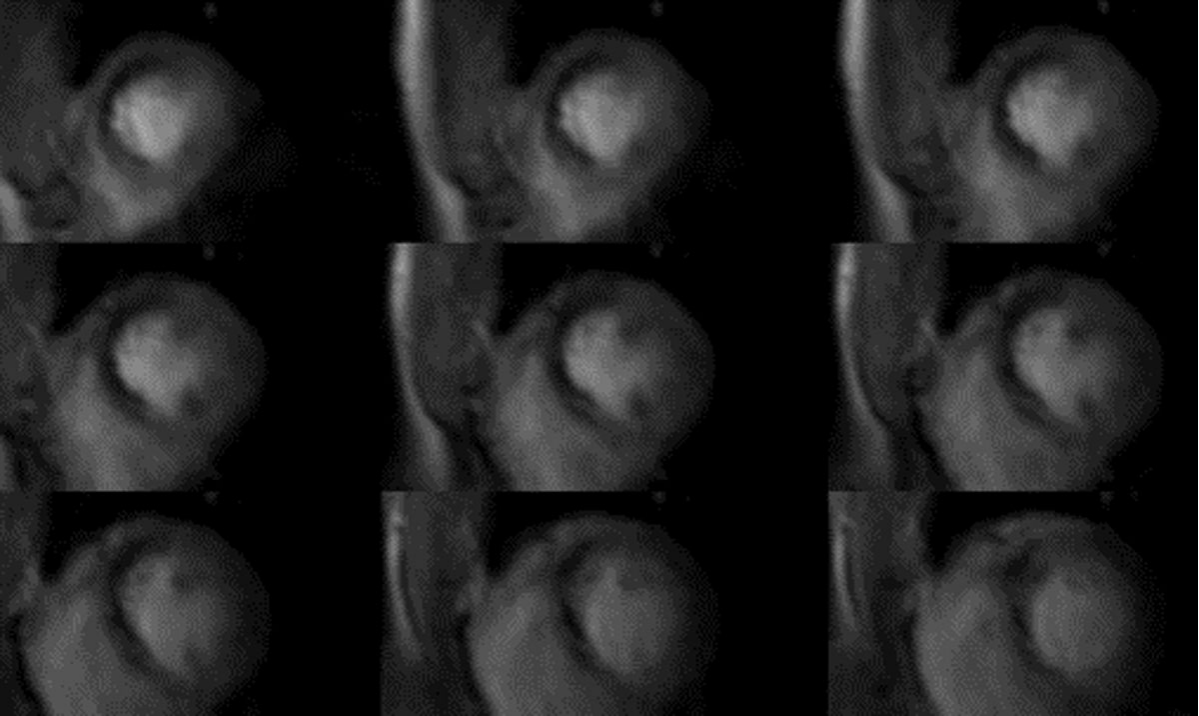
3-D CMR perfusion scans during adenosine stress showing a inducible perfusion deficit in anterior/anteroseptal segments extending from apical to basal slices. Invasive X-ray coronary angiogram demonstrated high-grade single-vessel disease with subtotal occlusion of the left anterior descending artery (LAD)
